# Separating the effects of mutation and selection in producing DNA skew in bacterial chromosomes

**DOI:** 10.1186/1471-2164-8-369

**Published:** 2007-10-12

**Authors:** Richard A Morton, Brian R Morton

**Affiliations:** 1 Department of Biology, McMaster University, 1280 Main Street West, Hamilton ON L8S 4K1, Canada; 2 Department of Biological Sciences. Barnard College, Columbia University, 3009 Broadway, New York NY 10027, USA

## Abstract

**Background:**

Many bacterial chromosomes display nucleotide asymmetry, or skew, between the leading and lagging strands of replication. Mutational differences between these strands result in an overall pattern of skew that is centered about the origin of replication. Such a pattern could also arise from selection coupled with a bias for genes coded on the leading strand. The relative contributions of selection and mutation in producing compositional skew are largely unknown.

**Results:**

We describe a model to quantify the contribution of mutational differences between the leading and lagging strands in producing replication-induced skew. When the origin and terminus of replication are known, the model can be used to estimate the relative accumulation of G over C and of A over T on the leading strand due to replication effects in a chromosome with bidirectional replication arms. The model may also be implemented in a maximum likelihood framework to estimate the locations of origin and terminus. We find that our estimations for the origin and terminus agree very well with the location of genes that are thought to be associated with the replication origin. This indicates that our model provides an accurate, objective method of determining the replication arms and also provides support for the hypothesis that these genes represent an ancestral cluster of origin-associated genes.

**Conclusion:**

The model has several advantages over other methods of analyzing genome skew. First, it quantifies the role of mutation in generating skew so that its effect on composition, for example codon bias, can be assessed. Second, it provides an objective method for locating origin and terminus, one that is based on chromosome-wide accumulation of leading *vs *lagging strand nucleotide differences. Finally, the model has the potential to be utilized in a maximum likelihood framework in order to analyze the effect of chromosome rearrangements on nucleotide composition.

## Background

With the recent accumulation of complete bacterial genome sequences there has been increased attention to prokaryote chromosome organization. One prominent aspect of most of these genomes is that several features, such as nucleotide composition and coding strand bias, display an organization that is centered on the origin of replication [1]. In these chromosomes, as exemplified by *Escherichia coli *[2,3], replication initiates at a single origin (Ori) and proceeds bi-directionally to a terminus (Ter) where the two forks meet [4]. This divides the chromosome into a replichore [5], defined as a chromosome with two oppositely replicated halves (or replication arms), within each of which there is a leading and lagging strand such that one DNA strand is leading within one replication arm but lagging within the other. Many bacterial genomes display a compositional asymmetry between the two DNA strands within a replication arm meaning that Parity Rule 2, which stipulates that the frequencies of A and T are equal as are the frequencies of G and C, is violated [6-8]. Observed strand asymmetry, or skew, in base composition is either a purine (G and A) or a keto (G and T) excess on one strand and the leading strand in one replication arm shows the same skew as the leading strand in the other despite the fact that they are opposite genome strands.

One issue that has arisen from these observations is the cause of the compositional asymmetry between strands, with evidence having been presented for contributions from both mutation and selection [1,9]. Many of the prokaryotes that have a replichore structure also have a bias towards coding genes, particularly 'essential' genes [1], on the leading strand [6,8,10-12] suggesting that composition asymmetry could result from selection and/or transcription-coupled mutation and repair processes [9,10,13]. There is also evidence, though, that leading and lagging strands differ in mutation bias [7,8,14], which has interesting and important implications for genome evolution [14,15]. Although there have been estimates of the contribution of mutation to skew in several genomes that suggest a role for selection [7] the issue has not been studied within a statistical framework.

Replichores with strand asymmetry have also been exploited to make inferences about the location of an origin of replication when the origin has not been mapped experimentally, which is the case in the vast majority of sequenced genomes. The compositional difference between leading and lagging strands, and the replichore structure in general, means that the two DNA strands have complementary composition biases in the two replication arms of these genomes. Plotting composition skew along a sliding window leads to a characteristic pattern in which the origin lies at a point where a given measurement of strand asymmetry switches between positive and negative values. This type of graphical approach has been used frequently to infer the location of replication origins [4,16-20] but these approaches have the disadvantage that determining the existence of skew and where it switches strand is subjective [9]. A more objective linear discriminant analysis has also been developed [21], but this method does not account for gene density nor does it utilize intergenic regions [9].

We develop a simple bipartition model (Methods) that exploits the existence of a replichore structure with strand asymmetry and provides for analyses within a maximum likelihood (ML) framework. In the current study we will apply the model to bacterial chromosomes (see Table 1) in two analyses. The first is the identification of two peaks on a likelihood surface, the locations of the putative origin and terminus of replication. Additional information, such as the organization of ribosomal RNA (rRNA) genes [22], can then be used to infer which of the two locations is the origin. In this study we assign the origin to maximize the arrangement of rRNA genes on the resulting leading strands [22] and then compare the location of our putative origin to "origin-associated" genes that are located at or close to the replication origin in certain genomes [23]. The second application is a statistical analysis of the contribution of mutation to nucleotide composition skew across bacterial species.

**Table 1:** Chromosomes analyzed from eubacterial phyla^1^

Phylum	Class^1^	Number
Actinobacteria		23
Bacteroidetes		5
Chlamydiae		11
Cyanobacteria		17
Deinococcus-Thermus		4
Firmicutes		79
	Mollicutes	16
	Lactobacillales	27
	Clostridia	7
	Bacillales	29
Proteobacteria		197
	Alphaproteobacteria	55
	Betaproteobacteria	38
	Deltaproteobacteria	11
	Epsilonproteobacteria	8
	Gammaproteobacteria	85
Spirochaetes		6
Others		10

^1^The two phyla with the largest sample sizes are shown sub-divided by Class representation.

## Results and discussion

We applied the bipartition model to 352 fully sequenced bacterial chromosomes. An assessment of the mutational (which we call R-dependent) component of compositional skew (see Methods) requires an identification of the origin and terminus of replication. Since these have not been empirically identified in most genomes we first use the model to generate a maximum likelihood estimation of the two loci and discuss the accuracy of this approach. Once the putative origin and terminus have been identified for each chromosome we use the model to quantify the degree to which the mutational difference between leading and lagging strands generates a skew.

### Replication arms comprise half of most bacterial chromosomes

**Figure 1:**
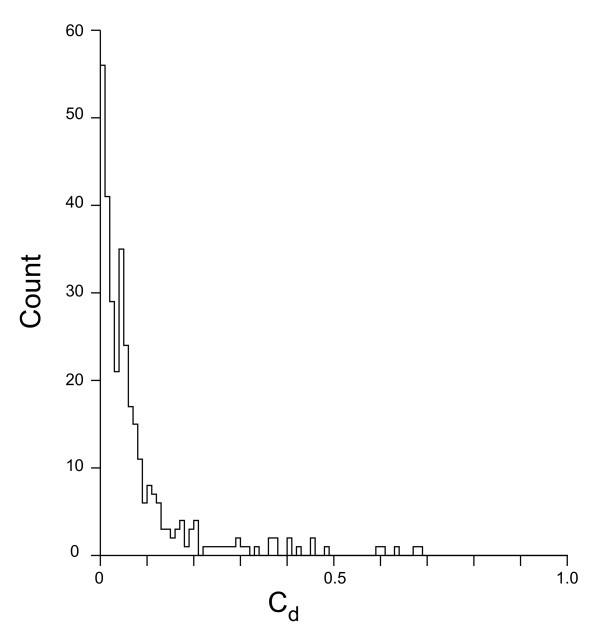
**Chromosome division**. The distribution of C_d _(chromosome division, Equation 10) based on [S_1_, S_2_]^ML ^pairs for the 326 chromosomes indicated in the text. C_d _is plotted along the X axis and represents the deviation from equal chromosome division.

We expect that if the unordered pair of sites identified by our model as the potential origin and terminus ([S_1_, S_2_]^ML^, see Methods) is an accurate estimation of the replication origin and terminus then these two sites should divide the chromosome roughly in half. (This chromosome division does not depend upon the assignment of S_1_^ML ^as an origin or terminus, which will be the subject of the next section.) The existence of such a physical balance is the basis of the Adopt-Adapt model [24,25,4], which proposes that this balance would guarantee a synchronous completion of replication by the two forks. The distribution of chromosome divisions, measured by the statistic C_d _(see Equation 10, Methods) is shown in Figure 1 for the 326 circular chromosomes in which the 95 percent confidence interval of C_d _was less than 20% of the chromosome. Linear chromosomes were also excluded since the method of calculating the confidence interval cannot be applied to them. We took C_d _between 0 and 0.2 as indicating a relatively equal division of chromosome arms and > 0.2 as an arbitrary measure of an inequitable division: these are chromosome in which [S_1_, S_2_]^ML ^generated a replication arm less than 40% of the total length. Of the 326 chromosomes analyzed, 31 (9.5%) had an inequitable division by our definition (see Table 2). Overall, this result agrees with the prediction of the 'Adopt-Adapt' model. The advantage to such an arrangement would presumably be that it results in the shortest possible replication time, which would otherwise be limited by the time taken to replicate the longer of the two arms.

**Table 2:** Chromosomes with unequal replication arms^1^

Taxon^2^	Accession	Phylum; Class^3^
*Acidobacteria bacterium *Ellin345	NC_008009	Acidobacteria
*Aquifex aeolicus *VF5	NC_000918	Aquificae
Aster yellows witches'-broom phytoplasma AYWB	NC_007716	Firmicutes; Mollicutes
*Bordetella parapertussis*	NC_002928	Proteobacteria; Betaproteobacteria
*Bordetella pertussis *Tohama I	NC_002929	Proteobacteria; Betaproteobacteria
*Burkholderia cenocepacia *AU1054	NC_008060	Proteobacteria; Betaproteobacteria
*Burkholderia thailandensis *E264	NC_007651	Proteobacteria; Betaproteobacteria
Candidatus *Blochmannia pennsylvanicus *str BPEN	NC_007292	Proteobacteria; Gammaproteobacteria
Candidatus *Pelagibacter ubique *HTCC1062	NC_007205	Proteobacteria; Alphaproteobacteria
*Desulfitobacterium hafniense *Y51	NC_007907	Firmicutes; Clostridia
*Francisella tularensi *subsp. *holarctica*	NC_007880	Proteobacteria; Gammaproteobacteria
*Haemophilus ducreyi *35000HP	NC_002940	Proteobacteria; Gammaproteobacteria
*Heliobacter hepaticus *ATCC 51499	NC_004917	Proteobacteria; Epsilonproteobacteria
*Idiomarina loihiensis *L2TR	NC_006512	Proteobacteria; Gammaproteobacteria
*Mycoplasma hyopneumoniae *J	NC_007295	Firmicutes; Mollicutes
*Mycoplasma mobile *163K	NC_006908	Firmicutes; Mollicutes
*Mycoplasms penetrans *HF2	NC_004432	Firmicutes; Mollicutes
*Nitrosomona europaea *ATCC 19718	NC_004757	Proteobacteria; Betaproteobacteria
*Prochlorococcus marinus *MIT9313	NC_005071	Cyanobacteria
*Pseudomonas aeruginosa *PAO1	NC_002516	Proteobacteria; Gammaproteobacteria
*Pseudomonas putida *KT2440	NC_002947	Proteobacteria; Gammaproteobacteria
*Shigella dysenteriae *Sd197	NC_007606	Proteobacteria; Gammaproteobacteria
*Silicibacter pomeroyi *Megaplasmid	NC_006569	Proteobacteria; Alphaproteobacteria
*Sinorhizobium meliloti *plasmid pSymA	NC_003037	Proteobacteria; Alphaproteobacteria
*Sodalis glossinidius *str. 'morsitans	NC_007712	Proteobacteria; Gammaproteobacteria
*Synechoccus elongatus *PCC7942	NC_007604	Cyanobacteria
*Thermus thermophilus *HB8	NC_006461	Deinococcus-Thermus
*Wolbachia ehdosymbiont *of *D. melanogaster*	NC_002978	Proteobacteria; Alphaproteobacteria
*Xylella fastidiosa *9a5c	NC_002488	Proteobacteria; Gammaproteobacteria
*Yersenia pestis *Antiqua	NC_008150	Proteobacteria; Gammaproteobacteria
*Yersenia pestis *MED	NC_005810	Proteobacteria; Gammaproteobacteria

^1^One replication arm less than 40% of the total chromosome length (see text).

^2^Primary chromosome unless otherwise indicated.

^3^Class is given only for Firmicutes and Proteobacteria as in Table 1.

Given an expectation for physical balance of chromosomes, it is possible that the 31 chromosomes with an inequitable division have undergone recent rearrangements. Large indels in either replication arm or inversions that include either the origin or the terminus could lead to deviations from equitable distribution. (Inversions that do not include the real replication origin could influence the ability to detect significant compositional skew but will not affect C_d_.) This cannot be investigated for all 31 cases since many chromosomes have not been studied in great detail or do not have close relatives for comparison, but evidence suggests that at least some of these chromosomes have undergone the predicted rearrangements. For example, *Pseudomonas aeruginosa *PA01 has a large inversion encompassing about 1/3 the genome [26], *Yersinia pestis *str. 91001 has significant inversions surrounding the putative origin [27] and *Prochlorococcus marinus *MIT9313 has several genome rearrangements relative to strain MED4 and is also much larger [28]. Candidatus *Blochmannia pennsylvanicus *str. BPEN has undergone significant gene loss relative to *B. floridanus *[29], *Halobacterium sp*. NRC-1 contains 91 Insertion Sequences from 12 different families [30] and Candidatus *Pelagibacter ubique *HTCC1062 has a very small genome that appears to have undergone numerous recent deletions [31]. These rearrangements suggest the possibility that the origin and terminus in these chromosomes do, in fact, yield a noticeably inequitable division. Although it seems likely that selection would favor an equitably divided genome, it is not known how rapidly a chromosome would regain equitable division following such a rearrangement and the genomes in Table 2 may be evolving towards a more equitable division. Alternatively, it could be that the genome assembly was performed incorrectly (see [32] for such an example) or that in these cases skew is not at equilibrium as a result of genome rearrangement and [S_1_, S_2_]^ML ^does not represent the origin and terminus. If this is the case then any method that utilizes composition skews to estimate the origin of replication, whether our ML approach or a graphical approach, would be misled.

### Locating the putative replication origins of bacterial chromosomes

We used [S_1_, S_2_]^ML ^to assign a putative origin/terminus pair (which we then call [P_O_, P_T_]^ML ^where P_O_^ML ^is either S_1_^ML ^or S_2_^ML ^and P_T_^ML ^is the other location) for the 352 chromosomes in our dataset. As described in the Methods we used several different methods to accomplish this; each are described separately. Where possible we can compare our results to empirically identified replication origins to assess the accuracy of our approach.

#### Linear chromosomes

The five linear chromosomes are relatively simple since an origin has been annotated for each at the center of the genome and these annotations appear to be reliable: the annotations for *Borrelia burgdorferi *and *B. garnii *are based on empirical evidence [17] as are the annotations for the two Streptomyces taxa [33], and in the case of *A. tumefaciens *the annotation is based on an analysis of the *repABC *genes [34]. Therefore, we can assign P_O_^ML ^and P_T_^ML ^for the linear chromosomes in the dataset using the annotated origins and assess our method. We find a putative origin near the annotated origin in each of the five linear chromosomes (Table 3); in three it is within 0.1% of the genome length of the annotated origin while in the other two it is within 3% of the genome length. In each case the other site is near the end of the chromosome. The data indicate that the bipartition model locates the origin and terminus accurately in these five chromosomes.

**Table 3:** Comparison of the origin of replication and the putative ML origin for each of the five linear chromosomes

Bacterial Species	Accession	Origin Signal^1^	Origin Location^1^	[S_1_^ML^, S_2_^ML^]^2^	P_o_^3^	Distance^4^
Streptomyces *avermitilis*	NC_003155	oriC	0.586	0.569, 0.994	0.569	0.017
Streptomyces *coelicolor*	NC_003888	oriC	0.493	0.463, 0.987	0.463	0.030
*Borrelia burgdorferi*	NC_001318	*dnaA-N*	0.503	0.000, 0.503	0.503	0.000
*Borrelia garinii*	NC_006156	*dnaA-N*	0.509	0.000, 0.510	0.510	0.001
*A. tumefaciens*^2^	NC_003305	*repA*	0.494	0.000, 0.493	0.493	0.001

^1^Location of the annotated origin as a fraction of chromosome length starting at the NCBI site 1.

^2^Location of [S_1_, S_2_]^ML ^pair as a fraction of chromosome length.

^3^P_O_^ML ^as inferred by which of S_1_^ML ^or S_2_^ML ^is closest to the annotated origin.

^4^Distance between the annotated origin and P_O_^ML^.

#### Ribosomal RNA genes in circular chromosomes

There is strong evidence that circular chromosomes are organized such that the ribosomal RNA (rRNA) genes tend to be located on the leading strand of replication regardless of where they are distributed along the length of the chromosome [22]. We used this as a basis for assigning [P_O_, P_T_]^ML ^in the 319 primary and 28 secondary circular chromosomes in our dataset. If S_1_^ML ^is assigned as the origin then in the chromosome portion S_1_^ML ^→ S_2_^ML ^the + strand is the leading strand while in the S_2_^ML ^→ S_1_^ML ^portion the – strand is leading. Assigning S_2_^ML ^as the origin reverses this leading strand assignment. We calculated the proportion of rRNA coded on the leading strand in the two possible organizations and assigned the origin based on which of the two resulted in a majority of rRNA genes on the leading strand. In 14 chromosomes there was no annotation of rRNA genes while in 8 others we could not assign an origin because both possibilities yielded 50% rRNA genes on each strand. For the remaining 325 chromosomes, 304 had 100% of the rRNA genes on the putative leading strand, 18 others had > 75% of the rRNA genes on the putative leading strand and the 3 others had 60% of the rRNA genes on the putative leading strand. An interesting point involves assignment of the origin to the middle of the linear *Borrelia *chromosomes. In *Borrelia garinii *only 20% of the rRNA genes are on the leading strand. The 23S rRNA genes in *Borrelia garinii *are inverted with respect to *Borrelia burgdorferi *where 97% of the rRNA sites are on the leading strand.

#### Evidence other than rRNA genes

For the 22 circular chromosomes noted above for which an origin could not be assigned based on the distribution of rRNA genes, we designated [P_O_, P_T_]^ML ^based on assigning the leading strand in each replication arm as the strand for which the replication-induced effect was inferred to make G > C at four-fold degenerate (D_4_) sites. The rationale for this is discussed in the Methods and necessarily limits the conclusions we can draw about composition bias in these chromosomes.

### Identification of genes consistently located near the origin of bacterial chromosomes

One interesting feature of the circular chromosomes in our dataset is the existence of genes that may function in segregating the two replication products during cell division [23] and which appear to be located near the origin of replication in many chromosomes. In this section we compare the locations of P_O_^ML ^in each chromosome to these genes in order to determine the degree of concurrence. We will do this separately for the 28 secondary and 319 primary circular chromosomes; the 5 linear chromosomes will not be analyzed in this section. This comparison is not essential for our identification of [P_O_, P_T_]^ML ^but it provides a way of both assessing our model as well as the proposal that these genes do tend to be located near the replication origin.

#### Secondary chromosomes

In the secondary chromosomes, the segregation system involves either the *parA *and *parB *genes, along with a co-localized *cis *element (*parS*), which is bound by the ParB protein, or the *repA, repB *(which are apparently *parA *and *parB *homologs respectively, see [35]) and *repC *genes. We will refer to this generally as *par/rep *system; it is found in all of the secondary chromosomes in this study. The location of the *par/rep *genes in each of the 28 secondary chromosomes was determined by annotation if possible, while for the chromosomes in which neither was annotated we used a BLAST search to locate the gene. In 26 of the 28 secondary chromosomes we find that P_O_^ML ^is within 1% of the genome length of *par/rep *and in one of the other two chromosomes the distances is 2.6% of the chromosome length (Table 4). In the remaining case, *Rhodobacter sphaeroides *chromosome 2, it is P_T_^ML ^and not P_O_^ML ^that is near *par/rep*, although the assignation of Po was based on it resulting in all of the rRNA genes being on the leading strand (see Additional file 1). Therefore, either P_O_^ML ^is near the *par/rep *location and none of the rRNA genes are on the leading strand or P_T_^ML ^is near the *par/rep *location and all of the rRNA genes are on the leading strand. It is possible that a recent inversion or other chromosome rearrangement resulted in a separation of the segregation genes and the origin, leading to an incorrect assignment of the origin based on rRNA distribution. Overall, the co-localization of P_T_^ML ^and the *par/rep *genes in all but this one case provides strong evidence that the bipartition model is accurately locating the replication origin.

**Table 4:** Comparison of the location of partition genes and the putative ML origin for each of the 28 secondary circular chromosomes

Bacterial Species	Accession	*parA/repA*^1^	P_O_^ML2^	Distance^3^
*Brucella abortus*	NC_006933	0.998	0.999	0.001
*Brucella melitensis, 16M*	NC_003318	0.079	0.079	0.000
*Brucella melitensis, Abortus 2308*	NC_007624	0.998	0.999	0.001
*Brucella suis*	NC_004311	0.998	0.999	0.001
*Burkholderia sp. 383, Chr 2*	NC_007511	0.002	0.000	0.002
*Burkholderia sp. 383, Chr 3*	NC_007509	0.996	0.001	0.005
*Burkholderia cenocepacia, Chr 2*	NC_008061	0.879	0.878	0.001
*Burkholderia cenocepacia, Chr 3*	NC_008062	0.315	0.320	0.005
*Burkholderia mallei*	NC_006349	0.997	0.001	0.004
*Burkholderia pseudomallei, 1710b*	NC_007435	0.576	0.578	0.002
*Burkholderia pseudomallei, K96243*	NC_006351	0.998	0.001	0.003
*Burkholderia thailandensis*	NC_007650	0.998	0.000	0.002
*Burkholderia xenovorans, Chr 2*	NC_007952	0.000	0.006	0.006
*Burkholderia xenovorans, Chr 3*	NC_007953	0.981	0.979	0.002
*Photobacterium profundum*	NC_006371	0.999	0.000	0.001
*Ralstonia eutropha*	NC_007348	0.775	0.778	0.003
*Ralstonia metallidurans*	NC_007974	0.943	0.946	0.003
*Ralstonia solanacearum*	NC_003296	0.001	0.002	0.001
*Rhodobacter sphaeroides*	NC_007494	0.996	0.513	0.483
*Silicibacter sp. TM1040*	NC_008043	0.517	0.519	0.002
*Silicibacter pomeroyi*	NC_006569	0.678	0.668	0.010
*S. meliloti, pSymA*	NC_003037	0.999	0.991	0.008
*S. meliloti, pSymB*	NC_003078	0.034	0.060	0.026
*Vibrio cholerae*	NC_002506	0.999	0.005	0.006
*Vibrio fischeri*	NC_006841	0.999	0.002	0.003
*Vibrio parahaemolyticus*	NC_004605	0.999	0.000	0.001
*Vibrio vulnificus CMCP6*	NC_004460	0.689	0.692	0.003
*Vibrio vulnificus, YJ016*	NC_005140	0.999	0.002	0.003

^1^*parA or repA *location (see text), as a fraction of chromosome length starting at the NCBI site 1.

^2^P_O_^ML ^as inferred from rRNA sites or σ_GC _> 0 (see text).

^3^Distance between *parA/repA *location and P_O_^ML^.

#### Primary chromosomes

For the analysis of the 319 primary, circular chromosomes we excluded 21 chromosomes for which the statistical significance of [S_1_, S_2_]^ML ^is uncertain; 14 of them because there was no evidence for a replication-induced effect on composition, and the other 7 because the confidence limit of the genome division parameter C_d _spanned more than 20% of the chromosome. For the remaining 298 chromosomes we used what we will call an "origin gene" approach to assess the location of P_O_^ML^, an approach that is similar to what was applied to the secondary chromosomes. Unlike the secondary chromosomes, there is no single cluster of genes that can be used to locate the origin in all of the primary genomes in the dataset. Despite this lack of a universal gene cluster, evidence indicates that the origin of replication in these primary chromosomes is frequently located nearby any or all of several genes which may actually have formed an ancestral origin gene "cluster" [36]. These genes, which we will refer to as "origin genes", are *parA*, *parB*, *gidA*, *gidB*, *yidC*, *yidD*, *rnpA*, *rpmH*, *dnaA *and *dnaN *[23]. These genes vary in location with respect to one another and thus cannot always be used to determine a single chromosome location, but despite this, evidence from chromosomes where these genes have been studied indicates that at least one of these genes is close to the origin in any given chromosome [23].

We used these so-called origin genes to assess P_O_^ML ^as follows. NCBI annotations were used to locate all genes annotated with any of the 10 gene names. Since these genes are not located together across all chromosomes we treated them as 5 pairs that tend to be co-located; *parA *and *parB*, *gidA *and *gidB*, *yidC *and *yidD*, *rnpA *and *rpmH*, and *dnaA *and *dnaN*. A chromosome was scored as having a specific gene pair if both genes in that pair were annotated and the mid-points were within 5 kb of each other or if only one gene of the pair was annotated (under the assumption that the second gene may not have been annotated). We then scored P_O_^ML ^as being located near that specific pair if it was within 1% of the chromosome length of the midpoint between the two genes or from the mid-point of the single annotated gene. Of the 298 chromosomes, 57 had none of the 10 genes annotated and thus could not be assessed. For the remaining 241, Table 5 shows the number of gene pairs that were scored for each chromosome along with how many chromosomes had P_O_^ML ^near any pair. Overall 154 chromosomes (63.9%) have the putative origin within 1% of the genome length of one of the pairs and of the 156 chromosomes that have 3 or more gene pairs, 115 (73.7%) have the putative origin within 1% of the length from a gene pair. Of the 9 chromosomes with all 5 gene pairs located, 8 have P_O_^ML ^near at least one pair and in the one exception (*Azoarcus *sp. EbN1), all 5 gene pairs were close to one another and the putative origin was between 5.1% and 6.0% of the pairs. In addition, in cases where there was more than one gene pair annotated, the most common category in Table 5 is that in which P_O_^ML ^is located within 1% of all identified gene pairs. This indicates that the origin genes were all clustered and very close to our putative origin. The results confirm that the replication origin is frequently near one or more of the set of "origin genes".

**Table 5:** Number of chromosomes in which the putative origin is located near one or more origin gene pairs

	Number of origin gene pairs near the putative origin^2^							
Gene Pairs^1^	0	1	2	3	4	5	> 0	Total^3^
0	57	-	-	-	-	-	-	57
1	24	7	-	-	-	-	7	31
2	22	11	21	-	-	-	32	54
3	17	3	23	24	-	-	50	67
4	23	11	12	3	31	-	57	80
5	1	2	0	1	0	5	8	9

^1^Number of "origin gene" pairs found in any given chromosome, as defined in the text.

^2^Number of chromosomes for which the specified number of origin gene pairs is found within 1% of the chromosome length from P_O_^ML^. When multiple origin gene pairs are found near P_O_^ML ^it indicates that the gene pairs are close together on the chromosome.

^3^Total number of chromosomes with that number of annotated gene pairs regardless of whether or not any pair is close to P_O_^ML^.

### R-dependent component of skew across bacterial chromosomes

Once we have identified [P_O_, P_T_]^ML ^for each chromosome we can use our model to calculate the effect of leading *vs *lagging strand mutation differences, the R-dependent component of skew. As discussed in the Methods, the R-dependent component is best estimated by using sites least affected by selection. The intergenic (IG) regions and four fold degenerate (D_4_) sites of CDS regions are possible choices. We used relative σ values (R_G _and R_T_, see Equation 9, Methods) to compare the R-dependent component of skew across all 352 bacterial chromosomes. These measures represent the proportional increase of G and T, respectively, on the leading strand as a result of mutational differences between the leading and lagging strands.

There is a strong correlation between IG and D_4 _for both R_G _(Slope = 0.68; R^2 ^= 0.76, P < < 10^-6^) and R_T _(Slope = 0.46; R^2 ^= 0.73, P < < 10^-6^), indicating that they give similar estimates of the relative contribution of the R-dependent mutation effect to skew. It also suggests that transcription-coupled mutation effects are not strongly affecting the estimates of R-independent effects at D_4 _sites. However, the data also show that for most chromosomes the absolute value of the skew is stronger for D_4 _sites than IG sites, for both R_G _and R_T_. We propose, based on the compact nature of intergenic sites in bacterial genomes and the existence of regulatory sequences within these regions, that this is most likely due to selective constraints on many intergenic sites similar to recent findings from Drosophila [37], and that the D_4 _sites provide the more accurate estimation of the contribution of the R-dependent component. Therefore, we will use these sites to examine this component of skew across bacterial chromosomes.

**Figure 2:**
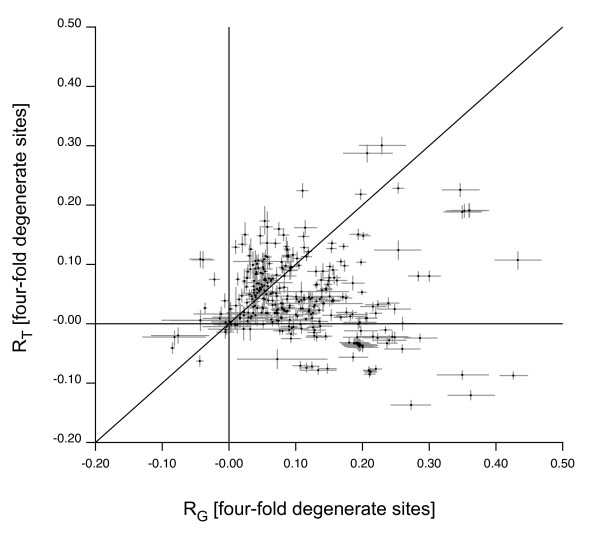
**Strand asymmetry across bacterial chromosomes**. A scatter plot of R_T _and R_G _for D_4 _sites on the leading strands of the 352 bacterial chromosomes. The values represent the average effect for the two leading strands in each of the replication arms, with the bars indicating the 95% uncertainty. The straight line represents R_T _= R_G_.

Given that there is also a strong correlation between D_4 _sites coded on the + and - strands across the 352 bacterial chromosomes (for R_G_, R^2 ^= 0.90; for R_T_, R^2 ^= 0.94), we calculated the average of the two strands for both R_G _and R_T _to obtain a single estimate to assess the relative strength of replication-induced skew (Figure 2). We also calculated a measure of overall skew SQRT(R_T_^2^+R_G_^2^) and those chromosomes with the strongest and weakest overall skew are shown in Table 6. There are 208 chromosomes that have a significantly stronger G/C than T/A skew (below the R_G _= R_T _line) and 90 that have a significantly stronger T/A than G/C skew (above the R_G _= R_T _line). R_G _and R_T _are not significantly different in the remaining 54. This suggests that mutation bias tends to generate a stronger G/C skew across microbial chromosomes [7] but a statistical analysis is confounded by the fact that the points are not independent due to shared ancestry as well as the fact that the species that have been sequenced have not been sampled randomly.

**Table 6:** Chromosomes with extreme skew

Species	Group	R_G_	R_T_	Skew^1^
Chromosomes with strongest overall skew				
Candidatus *Blochmannia floridanus*	Gammaproteobacteria	0.433	0.107	0.446
*Clostridium acetobutylicum *ATCC 824	Firmicutes	0.426	-0.087	0.435
*Ehrlichia canis str*. Jake	Alphaproteobacteria.	0.346	0.226	0.413
*Ehrlichia ruminantium *str. Welgevonden	Alphaproteobacteria	0.360	0.191	0.408
*Ehrlichia chaffeensis *str. Arkansas	Alphaproteobacteria	0.360	0.191	0.407
*Ehrlichia ruminantium *str. Gardel	Alphaproteobacteria	0.353	0.190	0.401
*Ehrlichia ruminantium *str. Welgevonden	Alphaproteobacteria	0.349	0.188	0.397
*Lactobacillus salivarius subsp. salivarius *UCC118	Firmicutes	0.362	-0.121	0.382
*Borrelia burgdorferi *B31	Spirochaetes	0.229	0.300	0.378
*Clostridium perfringens *str. 13	Firmicutes	0.350	-0.086	0.360
*Borrelia garinii *PBi	Spirochaetes	0.207	0.287	0.354
*Xylella fastidiosa *Temecula1	Gammaproteobacteria	0.254	0.228	0.341
*Bartonella quintana *str. Toulouse	Alphaproteobacteria	0.300	0.080	0.311
*Clostridium tetani *E88	Firmicutes	0.273	-0.137	0.305
*Bartonella henselae *str. Houston-1	Alphaproteobacteria	0.284	0.080	0.295
*Xylella fastidiosa *9a5c	Gammaproteobacteria	0.197	0.218	0.294
*Lactobacillus acidophilus *NCFM	Firmicutes	0.286	-0.024	0.287
*Buchnera aphidicola *str. Bp	Gammaproteobacteria	0.254	0.124	0.282
*Lactobacillus johnsonii *NCC 533	Firmicutes	0.260	-0.042	0.263
*Carboxydothermus hydrogenoformans *Z-2901	Firmicutes	0.260	0.000	0.260
Chromosomes with weakest overall skew				
*Corynebacterium efficiens *YS-314	Actinobacteria	0.023	0.019	0.030
*Nostoc sp*. PCC 7120	Cyanobacteria	0.028	0.005	0.028
*Sinorhizobium meliloti *1021 plasmid pSymA	Alphaproteobacteria	0.022	0.014	0.026
*Mycobacterium avium subsp. paratuberculosis *K-10	Actinobacteria	0.002	0.025	0.025
*Anaeromyxobacter dehalogenans *2CP-C	Deltaproteobacteria	0.022	-0.009	0.024
*Mycoplasma hyopneumoniae *232	Firmicutes	0.017	0.016	0.023
*Mycoplasma synoviae *53	Firmicutes	-0.012	0.016	0.020
*Wigglesworthia glossinidia*	Gammaproteobacteria	0.016	0.011	0.020
*Mycoplasma mycoides subsp. mycoides *SC str. PG1	Firmicutes	0.017	0.009	0.019
*Mycoplasma gallisepticum *R	Firmicutes	-0.014	0.009	0.016
*Aquifex aeolicus *VF5	Aquificae	-0.006	-0.014	0.015
*Mycoplasma hyopneumoniae *7448	Firmicutes	-0.012	-0.002	0.013
*Thermosynechococcus elongatus *BP-1	Cyanobacteria	-0.005	-0.010	0.012
*Synechocystis sp. PCC 6803*	Cyanobacteria	0.010	-0.002	0.010
*Synechococcus sp. JA-2-3B'a(2–13)*	Cyanobacteria	0.009	-0.001	0.009
*Baumannia cicadellinicola *str. Hc	Gammaproteobacteria	-0.003	-0.005	0.006
*Anabaena variabilis *ATCC 29413	Cyanobacteria	-0.005	0.003	0.006
*Mycoplasma pneumoniae *M129	Firmicutes	0.003	0.003	0.004
*Synechococcus sp. JA-3-3Ab*	Cyanobacteria	0.001	0.004	0.004
*Gloeobacter violaceus *PCC 7421	Cyanobacteria	0.003	-0.002	0.004

^1^Overall skew given by SQRT(R_G_^2 ^+ R_T_^2^)

The G/C skew is almost exclusively biased towards G, which agrees with the observation of Lobry and Sueoka [7] who found that G > C on the leading strand in almost all of the bacterial genomes they surveyed, the one exception being the linear chromosome of *Streptomyces coelicolor*. Since we used the criterion of G > C on the leading strand to assign P_O_^ML ^for 22 of the circular chromosomes, we excluded these from an assessment of the pattern of G/C bias. Of the remaining 330 chromosomes, only 9 show a significant leading strand skew towards C. These are *Bifidobacterium longum*, *Thermobifida fusca *YX, *Deinococcus radiodurans *R1, *Tropheryma whipplei *TW08/27, *T. whipplei *str. Twist, *Mycoplasma mobile *163K and *M. penetrans *HF-2 as well as the two linear *Streptomyces *chromosomes, *Streptomyces avermitilis MA-4680 *and *S. coelicolor*. We also find that among those chromosomes with a significant T/A skew (above or below the R_T _= 0 line), 245 have a significant skew towards T and 65 a significant skew towards A. Overall, there were 237 chromosomes with a significant G&T bias, 60 with a G&A bias, 5 with a C&T bias and 4 with a C&A bias on the leading strand. This analysis supports the finding of Lobry and Sueoka [7] that the G&T (keto) skew is more common than G&A (purine) skew on the leading strand. Statistical analysis is again confounded by non-independence of points.

**Figure 3:**
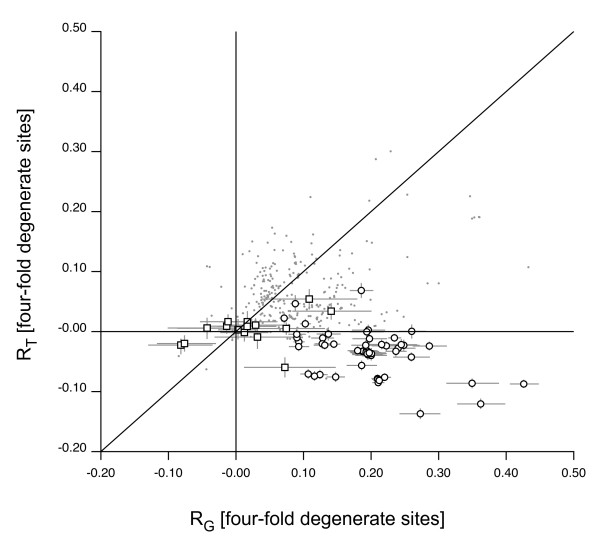
**Strand asymmetry in Firmicutes**. The same plot as in Figure 2 with the Firmicute chromosomes indicated as open points. Open squares indicate those of the class Mollicutes (which includes the Mycoplasma genus) while the open circles indicate all other Firmicutes.

Despite the limitations that arise from the non-independence of the chromosomes one conclusion that can be drawn from the data is that the chromosomes with a purine (G&A) skew are predominantly Gram-Positive bacteria (Figure 3). Lobry and Sueoka [7] previously noted the exception of the Gram-Positive *Lactococcus lactis *and *Staphylococcus aureus *to the general trend of keto skew. We show that it is a common feature across the Firmicutes, with the exception of the genus Mycoplasma with 12 representative chromosomes, all of which show very little overall R-dependent effect. Of the 64 genomes that have significant strand average skew towards A in Figure 2, 53 are Firmicutes and only 4 of the 79 sequenced Gram-Positive chromosomes show significant strand average skew towards T (*Mycoplasma penetrans *HF-2, *Bacillus subtilis subsp. subtilis *str. 168, *Bacillus licheniformis *ATCC 14580, and *Thermoanaerobacter tengcongensis *MB4). These trends are difficult to assess without a general knowledge of how rapidly mutational biases change and the degree of relationship in the genomes being compared, but it does appear that the Gram-Positive chromosomes tend to show a different general pattern of skew than other prokaryotes.

The difference in composition bias between the Firmicutes and the other bacterial lineages may be related to a general difference in the mode of replication. It has been noted previously that those species that have a leading strand bias towards A over T have a *polC *homolog in addition to a *dnaE *homolog [20]. These authors point out that proofreading in species with only a *dnaE *homolog involves an interaction of the Θ and α subunits while in species that also have a *polC *homolog proofreading involves only the α subunit. This difference is probably sufficient to result in the general difference in mutation pattern but it is also the case that the Firmicutes show a stronger tendency to code genes on the leading strand than do other lineages [1]. This could conceivably contribute to an R-dependent mutation effect if there is a transcription-coupled repair system or if the lagging strand is more frequently in the single stranded state as a result, given the much greater tendency of ssDNA than dsDNA to undergo cytosine deamination [1].

If we consider overall skew (Table 6) there are some general points that are apparent, although these might partly reflect the non-random sample of genomes that have been sequenced. The high degree of overall skew in Borrelia noted by Loby and Sueoka [7] is apparent in our data, and the other taxa they noted as having strong skew (*Treponema pallidum, Chlamydia muridarum and C. trachomatis*) rank in the 100 chromosomes with strongest skew (see Additional file 1). The Alphaproteobacteria (particularly the genera Ehrlichia, Gluconobacter, Bartonella and Brucella) and the Firmicutes are disproportionately represented in the high skew genomes (both make up 35% of the 20 chromosomes with highest skew while Alphaproteobacteria and Firmicutes are 15.6% and 22.4% of the sequenced chromosomes respectively) while the Cyanobacteria tend to have low skew (7 of the 17 sequenced Cyanobacteria are in the 20 chromosomes with lowest skew). There are exceptions in each case (e.g. the endosymbiont Alphaproteobacterium Wolbachia and the Mycoplasma species) suggesting that mutational biases can vary dramatically and, perhaps, rapidly across lineages. In a previous survey, Rocha [1] also reported a low general skew in Cyanobacteria which is consistent with our finding, but also reported a low skew in the Alphaproteobacteria. The difference in the later case could be due to our use of an estimate from putatively neutral sites instead of a general composition bias.

## Conclusion

The bipartition model allows us to quantify the contribution of the mutational difference between leading and lagging strands to nucleotide skew and also allows us to estimate the locations of the origin and terminus in chromosomes with bi-directional replication when the accumulation of skew is sufficiently strong. The model has several advantages over other methods of analyzing genome skew. First, using the model we can quantify the role of mutation in generating skew so that the effect on composition, for example codon bias, can be assessed. Second, it provides an objective method for locating origin and terminus that exploits composition bias. Finally, the model has the potential to be utilized in a maximum likelihood framework in order to analyze various aspects of genome structure, such as the effect of chromosome rearrangements on nucleotide composition.

## Methods

### Chromosomes analyzed

Primary chromosomes from 350 bacterial strains at the NCBI Genome Project website [38] as of June 20 2006 were downloaded as were 29 secondary chromosomes chosen from these species on the basis of annotation. Given the evidence for multiple origins of replications in Archaeabacetria chromosomes [39-41] the 27 chromosomes, which include one secondary chromosome, annotated as Archaea were removed to give a total of 352 chromosomes studied. Of this total, 5 chromosomes are linear and all others are circular. (Two chromosomes were incorrectly annotated by the NCBI file as linear; *Baumannia cicadellinicola *str. Hc NC_007984  [42], and *Staphylococcus aureus susp. aureus *NCTC8325 NC_007795  [43]. Linear chromosomes were analyzed in the same manner since they can also be divided into two replication arms. The taxonomic distribution of these 352 chromosomes is given in Table 1 and summary data are provided (see Additional file 1).

## The bipartition model

We first develop a bipartition model that is the basis for ML methods. After deriving the core equations of the model we will discuss two specific applications of the model; an inference of the replication origin and terminus and an analysis of mutational contribution to skew. The approach of [7] to estimate the contributions of selection and mutation to skew is a special case of our model (see below). After developing the model we will illustrate it by application to *Escherichia coli *strain K12.

### R-dependent and R-independent components of skew

The nucleotide substitution process can in principle be divided into what we will call R(eplication arm)-dependent and R(eplication arm)-independent components. The R-dependent component is defined as whatever effect is generated due to mutational differences between leading and lagging strands while the R-independent component is comprised of those factors, mutational or selective, that affect substitutions in the same manner regardless of which strand is being considered. This latter definition includes any selective pressure at a given site as long as this selective pressure does not depend upon which of the two strands happens to be the coding strand (i.e., if switching the coding strand of a gene does not affect selective pressures for nucleotides on the coding strand). However, if we want to use the model to measure the contribution of the R-dependent component to compositional skew, it is important to note that when there is an inequitable distribution of coding genes and/or regulatory elements on the leading strand, there can be compositional skew even in the absence of an R-dependent component. This potential effect of selection and/or other transcription-coupled effects will be considered in specific applications of the model.

Given this separation of the substitution process, we can express the frequency of nucleotide *i *at any given site on a specified strand (e.g., the + strand of the NCBI annotation) of the genome by Equation 1 where π_i _represents the contribution of the R-independent and σ_i _the R-dependent factors respectively.

*f*_i _= π_i _+ σ_i _

Although it is not possible to calculate π and σ separately for each nucleotide, this division does allow us to estimate R-dependent and R-independent contributions to overall compositional skew (A-T and G-C differences). A circular chromosome that is replicated bi-directionally from a single origin is divided into two replication arms, each with its leading and lagging strands. We designate the annotated strand as + and define P as the arm in which this strand is the leading strand and ρ as the arm within which it is the lagging strand. The R-dependent factors within P affecting the + strand are given by σ^L^_i _while within ρ they are given by σ^l^_i _where L and l refer to the leading and lagging strand respectively. Since σ^L ^and σ^l ^are complementary (for example, σ^L^_A _= σ^l^_T_), R-dependent components can be represented throughout the genome using only the σ^L ^parameters, which from hereon will be written without a superscript designation. We can then write the frequencies of nucleotide *i *on the + strand within a replication arm (i.e. P_i _or ρ_i_) as in Equations 2a and 2b where *i *refers to any of A, C, G or T and *j *to the complementary nucleotide. Note that the R-dependent parameter (σ_i_) changes to the complementary nucleotide (σ_j_) between leading and lagging strands.

P_i _= π_i _+ σ_i _

ρ_i _= π_i _+ σ_j _

Given our partition of substitution dynamics, the A+T composition (C_A+T_) of the + strand within either replication arm can be written as Equation 3, with a similar equation for C_G+C _(C_G+C _= 1 – C_A+T_).

C_A+T _= π_A _+ π_T _+ σ_A_+ σ_T _

The skew parameters are given in Equations 4a and 4b where π_ij _represents the R-independent and σ_ij _the R-dependent components of compositional skew.

π_AT _= π_A _- π_T_   π_GC _= π_G _- π_C _

σ_AT _= σ_A _- σ_T_   σ_GC _= σ_G _- σ_C _

In the absence of selection and transcription-coupled effects, π_ij _= 0 and σ_ij _represents the skew generated by mutational differences between the leading and lagging strands.

We can now express the nucleotide frequencies for the + strand from Equation 1 in terms of C_A+T _and the skew parameters. For the P replication arm these are given in Equations 5a through 5d and similar equations hold for ρ.

P_A _= (1/2)(C_A+T _+ π_AT _+σ_AT_)

P_T _= (1/2)(C_A+T _- π_AT _- σ_AT_)

P_C _= (1/2)(C_G+C _- π_GC _- σ_GC_)

P_G _= (1/2)(C_G+C _+ π_GC _+ σ_GC_)

The values C_A+T_, π_AT _and σ_AT _can now be rewritten as in Equations 6a through 6c.

C_A+T _= (1/2) [(P_A _+ P_T_) + (ρ_A _+ ρ_T_)]

π_AT _= (1/2) [(P_A _- P_T_) + (ρ_A _- ρ_T_)]

σ_AT _= (1/2) [(P_A _- P_T_) - (ρ_A _- ρ_T_)]

The parameters for GC content and skew (C_G+C_, π_GC _and σ_GC_) can be calculated in a similar manner. These equations (6a, 6b and 6c) represent the core of our model for a chromosome with two replication arms since they allow us to estimate the R-independent (π) and R-dependent (σ) components of skew from nucleotide composition within each replication arm. Note that although we have been discussing circular chromosomes, the model also applies to linear chromosomes with bi-directional replication from a single origin. This is the general model that is implemented below in ML applications.

### Classifying sites to minimize effects of selection

As already noted, selective pressures for amino acid composition can contribute to skew, particularly if there is a coding strand bias in the genome [8]. Since these pressures are part of the R-independent component, if we want to measure the R-dependent component of skew then we will need to apply our model to neutral sites. This is possible since Equations 5 & 6 can be applied to any subset of sites within a chromosome. Therefore, if we use only the composition of relatively neutral sites, such as fourfold degenerate sites or intergenic sites, we can use the parameter σ determined from them as an estimate of the R-dependent component of skew. Nucleotide sites in each genome were classified based on the NCBI annotation according to codon position (C_1_, C_2_, C_3 _for CDS-coding genes) on both + and - strands, as RNA-coding or as intergenic (IG). Third codon position sites were further sub-classified as D_4 _if four-fold degenerate (this did not include those that are from a codon coding a six-fold degenerate amino acid).

### Maximum likelihood Implementation

Equations 6a-c (and similar ones for GC) allow an estimation of model parameters directly from nucleotide composition parameters within replication arms. The model can also be implemented in a ML framework using a variety of specific methods, discussed separately below, that differ in the constraints they introduce. A comparison of different methods will allow us to assess the explanatory power of the constraints. To implement these ML methods, the genome is divided into two replication arms. (This can be achieved either by using an annotated [Ori, Ter] or the putative locations determined using the method we will describe below.) This genome division defines the number of sites of each type within each replication arm such that there are M^k^_i _sites of type k that are nucleotide *i *in region P.

Expected nucleotide frequencies are calculated from the model parameters according to equations 5a-d (with corresponding equations for the ρ replication arm). We can then calculate the likelihood of the model correctly predicting the observed nucleotide composition of both replication arms. For site type k within the P replication arm, this likelihood is given by Equation 7a.

LPk=∏i(Pik)Mik
 MathType@MTEF@5@5@+=feaafiart1ev1aaatCvAUfKttLearuWrP9MDH5MBPbIqV92AaeXatLxBI9gBaebbnrfifHhDYfgasaacH8akY=wiFfYdH8Gipec8Eeeu0xXdbba9frFj0=OqFfea0dXdd9vqai=hGuQ8kuc9pgc9s8qqaq=dirpe0xb9q8qiLsFr0=vr0=vr0dc8meaabaqaciaacaGaaeqabaqabeGadaaakeaacqqGmbatdaqhaaWcbaGaeeiuaafabaGaee4AaSgaaOGaeyypa0ZaaebuaeaadaqadaqaaiabbcfaqnaaDaaaleaacqqGPbqAaeaacqqGRbWAaaaakiaawIcacaGLPaaaaSqaaiabbMgaPbqab0Gaey4dIunakmaaCaaaleqabaGaeeyta00aa0baaWqaaiabbMgaPbqaaiabbUgaRbaaaaaaaa@3ED0@

The likelihood for the replication arm P is then the product of the likelihood of each site type (Equation 7b). L_ρ _can be calculated in the same manner and the overall likelihood is the product of L_P _and L_ρ _.

LP=∏kLPk
 MathType@MTEF@5@5@+=feaafiart1ev1aaatCvAUfKttLearuWrP9MDH5MBPbIqV92AaeXatLxBI9gBaebbnrfifHhDYfgasaacH8akY=wiFfYdH8Gipec8Eeeu0xXdbba9frFj0=OqFfea0dXdd9vqai=hGuQ8kuc9pgc9s8qqaq=dirpe0xb9q8qiLsFr0=vr0=vr0dc8meaabaqaciaacaGaaeqabaqabeGadaaakeaacqqGmbatdaWgaaWcbaGaeeiuaafabeaakiabg2da9maarafabaGaeeitaW0aa0baaSqaaiabbcfaqbqaaiabbUgaRbaaaeaacqqGRbWAaeqaniabg+Givdaaaa@3762@

#### Method M_obs_

Model parameters for each site type are calculated according to Equations 6a-c (and corresponding equations for GC) using the observed nucleotide frequencies within each replication arm. The likelihood is not maximized. No constraints are introduced but sites are classified into 7 types: intergenic (IG), first codon position (C_1_), second codon position (C_2_) and third codon position (C_3_). The latter three are further divided into + and - strand (+ strand being defined by the NCBI file) to yield C_1_^+^, C_1_^-^, C_2_^+^, C_2_^-^, C_3_^+ ^and C_3_^-^. RNA coding sites are ignored, as are sites that are ambiguous in the NCBI annotation.

#### Method M_0_

This method uses the same approach and site classification as M_obs_. An initial guess for site parameters was obtained from M_obs _and maximum likelihood parameters were obtained using a simplex algorithm [44]. Each site type is optimized independently. There are 5 DF for each site type giving a total of 35 DF.

#### Method M_1_

This method introduces the constraint that the π parameters calculated for coding sites on the two chromosome strands, such as for example C_1_^+ ^and C_1_^-^, be complementary (π^+^_ij _= -π^-^_ij_). This constraint allows us to assess whether or not there is a significant difference between coding sites on the two strands using a likelihood ratio test. Thus there is only a single set of 5 parameters for each of the 3 CDS site types and 5 for IG sites, yielding 20 DF. An initial guess for these 20 parameters was obtained from M_obs _and then the values that maximized the total likelihood were obtained using the simplex algorithm.

#### Method M_2_

This method has the same constraints as the M_1 _method with the additional constraint that the σ_AT _and σ_GC _parameters are equal across all site types. Therefore, if mutational biases are consistent across sites, then comparing this method to an unconstrained method allows us to assess whether or not selection significantly affects our measurement of the R-dependent component of skew. There are 14 DF in this method (3 for IG and each of the 3 CDS types, plus a single σ_AT _and σ_GC _for all site types). Parameters were calculated as described for M_1_.

#### Method M_3_

This method removes the site classification such that all nucleotide sites in the chromosome are assumed to be equivalent. It retains the constraint that parameters for the two strands be complementary. This allows us to assess the value of site classification. This method has 5 DF and values were obtained as described for M_1_.

### Application to the *E. coli *K12 chromosome

The effects of constraints using the ML methods will be illustrated by application to the *E. coli *K12 chromosome. We divided this chromosome into two replication arms based on the annotated origin/terminus sites at approximately 3,924,000 and 1,589,000 respectively in the NCBI file and used Equations 5–7 for C_1 _[+/-], C_2 _[+/-], C_3 _[+/-] and intergenic (IG) sites to determine the likelihood of method M_obs_. The likelihood obtained from mean nucleotide composition within each replication arm (M_obs_) does not differ appreciably from that made according to ML (Table 7). Methods M_1 _and M_2 _produce significant decreases in likelihood, indicating that selection significantly affects our measure of the R-dependent component of skew.

**Table 7:** Likelihood comparisons for the different methods when applied to the *E. coli *K12 chromosome CDS and intergenic sites

Method	Log (L)	-2 × Diff^1^	Probability (DF_1_, DF_2_)^2^
M_obs_	- 2696055.16	NA	NA
M_0_	- 2696052.54	reference	NA
M_1_	- 2696075.49	45.9	5.5 × 10^-5 ^(35, 20)
M_2_	- 2696239.16	373.2	< < 10^-6 ^(35, 14)
M_3_	- 2740719.34	89,334	< < 10^-6 ^(35, 5)

^1^Relative to M_0_.

^2^Probability of the chi-square test with (DF_1 _- DF_2_) degrees of freedom.

The R-independent parameters (π) for coding sites show the expected complementarity between the + and - strands (Table 8), meaning that the π value calculated for the sites coded on one strand is the negative value of the π value for the sites coded on the other strand (since both parameters are calculated from the composition of the strand given in the NCBI file). The exception is the C_2 _site class for which the two strands show significantly different estimates of π_AT_, even accounting for the complementarity, suggesting a different average composition of proteins coded on the two strands. For IG sites, the π_AT _and π_GC _parameters are not significantly different from zero indicating that there is no net R-independent skew across the genome. This lack of an R-independent skew effect on IG sites, however, does not necessarily indicate a lack of selection; since we cannot assign a strand to intergenic sites, any selective effect could be equally distributed across the two strands with the result that we observe no net skew.

**Table 8:** Model parameters for the *E. coli *K12 chromosome when the M_O _method is implemented

Site Class^1^	C_A+T_^2^	π_AT_	σ_AT_	π_GC_	σ_GC_
IG	**0.578 **[0.576,0.579]	**0.0012 **[-0.001,0.003]	**- 0.0053 **[-0.007,-0.003]	**0.0015 **[-0.0004,0.003]	**0.0180 **[0.016,0.020]
C_1_^+^	**0.411 **[0.410,0.413]	**0.0944 **[0.093,0.096]	**- 0.0023 **[-0.003,0.0002]	**0.104 **[0.102,0.106]	**0.0072 **[0.007,0.010]
C_1_^-^	**0.409 **[0.408,0.410]	**- 0.0954 **[-0.097,-0.094]	**- 0.0010 **[-0.003,0.0004]	**- 0.107 **[-0.108,-0.105]	**0.0109 **[0.0092,0.013]
C_2_^+^	**0.593 **[0.592,0.594]	**- 0.0168 **[-0.018,-0.015]	**0.0079 **[0.0059,0.010]	**- 0.0461 **[-0.048,-0.045]	**0.0045 **[0.003,0.006]
C_2_^-^	**0.594 **[0.593,0.595]	**0.0133 **[0.011,0.015]	**- 0.0019 **[-0.004,0.001]	**0.0449 **[0.043,0.046]	**0.0038 **[0.002,0.005]
C_3_^+^	**0.442 **[0.441,0.444]	**- 0.0782 **[-0.080,-0.077]	**- 0.0056 **[-0.008,-0.005]	**0.0219 **[0.0193,0.0234]	**0.0249 **[0.022,0.026]
C_3_^-^	**0.439 **[0.438,0.441]	**0.0813 **[0.080,0.082]	**- 0.0064 **[-0.008,-0.005]	**- 0.0198 **[-0.021,-0.018]	**0.0241 **[0.022,0.026]
D4^+^	**0.381 **[0.378,0.383]	**-0.0548 **[-0.058,-0.052]	**-0.0079 **[-0.010,-0.005]	**0.0228 **[0.020,0.026]	**0.0464 **[0.043,0.049]
D4^-^	**0.380 **[0.387,0.382]	**0.0619 **[0.059,0.064]	**-0.0079 **[-0.010,-0.005]	**-0.0243 **[-0.027,-0.021]	**0.0421 **[0.039,0.046]

^1^Sites are classified as intergenic (IG) or coding (C) with the latter further classified by codon position, as indicated by the subscript, and chromosome strand, as indicated by the superscript. D_4 _are four fold degenerate C_3 _sites.

^2^The ML value is given with the 95% interval given in brackets.

There is a significant R-dependent effect (σ) for IG sites. The σ values are very similar for + *vs *- strands within the C_1 _and C_3 _site types, but second position codon sites (type C_2_) have significantly different estimates of σ_AT_. This difference indicates that selective constraints on the sites of the C_2 _class are not distributed equitably across the two leading strands of the genome. As with the different π values at C_2 _sites this suggests that there is a difference in protein composition between + and - strands, but it also suggests that there is a preference for coding certain types of genes (that differ in average composition) on the leading strand.

Overall, the results from this analysis of *E. coli *provide two specific points that are relevant to the analysis of other chromosomes. First, the method with the fewest constraints (M_0_) provides the best fit so we will use this method in our large-scale analyses. Second, given the influence of selection on estimates of σ_AT _and σ_GC_, we must limit the analysis to relatively neutral sites in order to separate the mutational contribution from selection. Two possible choices are intergenic (IG) sites and four-fold degenerate (D_4_) CDS sites and both of these will be utilized and compared in our applications of the bipartition model.

### Estimating the R-dependent component of composition skew

Lobry and Sueoka [7] introduced a graphical method for estimating the contributions of selection and mutation to composition skew. This involves plotting the T/A skew (i.e., [*f*_T _- *f*_A_]/[*f*_T _+ *f*_A_]) against the G/C skew for third codon (i.e., putatively neutral) sites of each gene in a genome and calculating mid-points for leading and lagging strand genes. The length of the line connecting these two points (their B_I _parameter) is an estimate of the role of mutational bias to skew while the distance from the (0, 0) point to the midpoint of this connecting line (their B_II _parameter) is an estimate of the contribution of selection [7].

The parameters derived from our model can be used to estimate these two parameters as summarized in Equations 8a and 8b. They only requires the determination of the unordered pair [S_1_, S_2_]^ML ^since B_I _and B_II _are invariant if the origin and terminus are interchanged.

B_I _= SQRT ((σ_AT _/C_A+T_)^2 ^+ (σ_GC _/C_G+C_)^2^)

B_II _= SQRT ((π_AT _/2C_A+T_)^2 ^+ (π_GC _/2C_G+C_)^2^)

However, there are some disadvantages to this graphical approach that our model can improve on. One is that it weights genes equally regardless of length, another is that the statistical confidence of the parameters is unclear as is the biological meaning of the values obtained. Most importantly, it does not allow us to estimate the contribution of mutation to G/C and T/A skew separately. Using the bipartition model we can make a direct estimate of the relative contributions of R-dependent (σ) effects to the composition of G and T (since a keto skew is most commonly observed) for each type of site that can be classified. The measures we propose are R_G _and R_T _as given in Equations 9a and 9b.

R_G _= σ_GC _/(C_G+C _- π_GC_)

R_T _= - σ_AT _/(C_A+T _- π_AT_)

At selectively neutral sites, and assuming that there are no transcription-coupled effects, these two values represent the fractional increase (or decrease) in the G and T compositions on the leading strands as a result of a difference between leading and lagging strand mutation bias. Thus, the model provides a statistical framework to assess significance and to easily compare mutational effects across genomes. We calculated R_G _and R_T _for each bacterial chromosome from Equations 9a and 9b (on the leading strand) based on the assignation of [P_O_, P_T_]^ML ^as described. To use sites that we thought would be relatively neutral we used model M_0 _parameters for intergenic (IG) and for C_3 _sites that coded for a four-fold degenerate amino acid (D_4_). Since parameters for D_4 _sites were determined separately for both plus and minus strands the values from the two strands were averaged for an overall estimate of the R-dependent effect.

### Estimation of parameter uncertainty

The 95% uncertainty range in model parameters and in R_T _and R_G _was estimated using the Metropolis-Hastings rejection sampling Monte Carlo algorithm [45,46] as follows. First, the ML vector of parameters, with Likelihood L_0_, was determined by the simplex algorithm. Next, one parameter was altered using a Gaussian random number with mean 0 and standard deviation σ which is input arbitrarily to start. The Likelihood for the new vector, L_1_, was calculated and the new vector "accepted" if L_1 _> L_0 _or with probability L_1_/L_0 _if L_1 _< L_0_. A burn-in phase involved repeating the acceptance process in sets of 100 with successively decreasing σ values until the average acceptance rate of new vectors is 50% over the 100 trials. After the burn-in was complete, the final burn-in vector of parameters and σ value were used to generate 10,000 parameter vectors by altering parameter values as above. The 10,000 were sorted for each parameter and the 95% range determined from the sorted set.

### Application to the *E. coli *K12 chromosome

Estimates of R-dependent effects, R_G _and R_T _were calculated for D_4 _and IG sites from the data in Table 8. For D_4 _sites in *E. coli *K12 the M_0 _model gives R_G _= 0.0716 (+ strand = 0.0778; - strand = 0.0653) and R_T _= 0.0215 (+ strand = 0.0181; - strand = 0.0248), while for IG sites the values are R_G _= 0.0428 and R_T _= 0.009. These results indicate that R-dependent effects increase the content of G at D_4 _sites on the leading strand by about 7% relative to the composition in the absence of R-dependent effects (decreasing C by the same amount) and the content of T at these sites by about 2%.

### ML determination of replication arms based on composition skew

The bipartition model can be used to infer the replication arms in genomes with a replichore structure by maximum likelihood since it provides a probability for each of the four nucleotides. This will be important for applications of our model to genomes for which an origin and terminus have not been annotated but it also introduces a more formal approach than skew plots to estimating these two loci.

Two chromosomal locations, S_1 _and S_2 _(which need not divide the chromosome into equal halves), define two replication arms, P and ρ, and thus determine the number and kind of sites within each. The likelihood of such a division is calculated according to model M_obs _for C_1 _[+/-], C_2 _[+/-], C_3 _[+/-] and IG sites. The likelihood for all possible unordered [S_1_, S_2_] pairs determines the global ML (designated here as [S_1_, S_2_]^ML^). Ideally we would calculate the likelihood for every possible [S_1_, S_2_] division in order to determine [S_1_, S_2_]^ML ^but given computational constraints we utilized the following strategy. A rough, initial likelihood map is made by computing log likelihood values over a 50 × 50 grid on the [S_1_, S_2_] plane. The maximum of this rough map was then used as the initial point to maximize the likelihood using the same simplex algorithm used for parameter optimization [44]. From [S_1_, S_2_]^ML ^we calculate the chromosome division statistic C_d _by Equation 10, in which min(*f*_1_, *f*_2_) is the minimum of the two chromosome fractions generated by S_1_^ML ^and S_2_^ML^. The C_d _statistic can range from 0, in cases where there is equitable division of the genome, to 1, in cases where the origin and terminus are at the same chromosome location yielding essentially a single replication arm.

C_d _= (0.5 - min(*f*_1_, *f*_2_))/0.5

A visualization of the ML surface and estimates of uncertainty in C_d_, S_1 _and S_2 _(as well as model parameters, see below) were obtained by the Metroplois-Hastings rejection sampling Monte Carlo algorithm, using a random walk from [S_1_, S_2_]^ML^. Proposal parameter (S_1 _and S_2_) values were obtained by a normally distributed random step of zero mean and a standard deviation that was chosen after a burn-in series to give an approximately 50% acceptance rate. If the proposed parameters improved the likelihood they were accepted. Otherwise, they were accepted with a randomly generated probability equal to the likelihood ratio. Ten thousand accepted parameter sets were sorted to give a 5-to-95 percentile range.

### Application to the *E. coli *K12 chromosome

**Figure 4:**
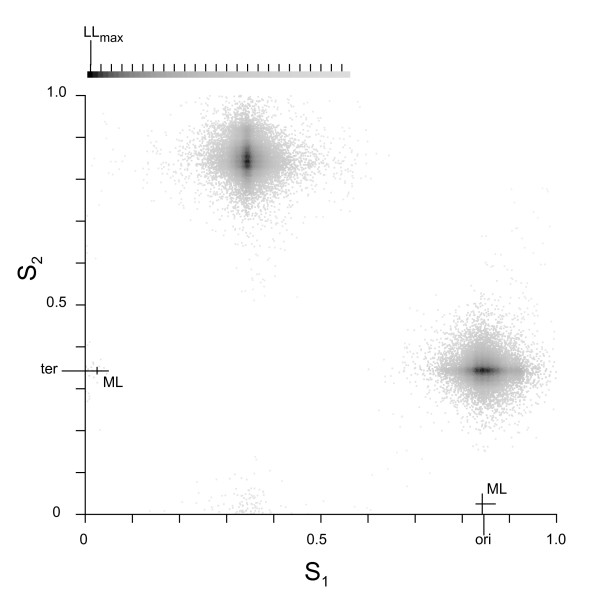
**Likelihood surface for the *E. coli *K12 chromosome**. This representation of the likelihood surface of the *E. coli *K12 chromosome (NC_000913) is based on the M_OBS _model (see Methods). The two axes represent relative chromosome length so that every point represents the likelihood analysis on the pair of chromosome locations [S_1_, S_2_]. The grey scale inset shows the conversion of log likelihood to grey value, with the maximum log likelihood (LL_max_) as black and the bars indicating -10 decrements. Since not every pair of chromosome locations was sampled the points are dispersed. The likelihood analysis is symmetrical around the diagonal so the two maxima are identical and represent just one pair of chromosome locations, but interchanging leading and lagging strands. The negative lines represent the location of the annotated ori and ter. The positive lines represent the maximum likelihood (ML) values and Monte Carlo estimated 95% ranges.

The replication origin for E. coli K12 has been located empirically and provides a good demonstration and test of our ML implementation. The log likelihood surface (see Figure 4) for the *E. coli *K12 chromosome (NC_000913) shows the two symmetrical peaks that differ in assignment of leading *vs *lagging strands as expected for the bipartition model. S_1_^ML ^was identified (correctly) as the putative origin by the distribution of rRNA sites. Thus, [P_O_, P_T_]^ML ^= [0.842, 0.342], where the locations are given as a fraction of the chromosome length (4,639,675) starting at the NCBI site 1. These two fractions represent the approximate nucleotide positions 3,908,000 and 1,589,000. The 95% intervals are [0.828 – 0.871, 0.334 – 0.352] for P_O_^ML ^and P_T_^ML^. The *oriC *and terminus (*dif *site) locations in *E. coli *are annotated at roughly positions 0.846 (3,924,000) and 0.342 (1,589,000) respectively in the NCBI file. The annotated origin and terminus both lie within the confidence interval and are very close to the ML estimates. Therefore, the bipartition model provides an accurate estimation of the origin of replication in this chromosome. Four additional examples are provided as supplemental material (see Additional file 2).

### Identification of the putative origin and terminus of replication

The likelihood surface has two peaks because exchanging S_1_^ML ^and S_2_^ML ^yields complementary σ parameters but the same likelihood. Thus, independent information must be used to determine a putative origin and terminus pair. We assigned the origin in one of several ways. For the 5 linear chromosomes we made use of the annotated origins in the NCBI files, which are all located near the center of the chromosome, by assigning whichever of S_1_^ML ^or S_2_^ML ^was closest to the annotated origin. For circular chromosomes we used annotated ribosomal RNA (rRNA) genes, where available, which are organized such that they are predominantly on the leading strand of replication across microbial genomes [22]. A third method for assigning the origin was utilized in the 14 chromosomes for which the rRNA annotation was not available and the 8 chromosomes for which there was an equal division of rRNA genes between the leading and lagging strands. In these cases we assigned the leading strand in each replication arm as the strand for which σ_GC _> 0 at fourfold degenerate sites (σ is defined for the leading strand). This use of σ_GC _> 0 as the criterion is based on the identification by Lobry and Sueoka [7] that, in the vast majority of bacterial chromosomes they studied, the leading strand has a skew of G > C. This last approach means that we are limited to knowing whether or not we find a G&T or G&A bias on one strand (and thus C&A or C&T on the other) in these particular chromosomes without definitively assigning the skew to leading or lagging strand. Regardless of the method used, the result is that we define either S_1_^ML ^or S_2_^ML ^as the putative origin, now designated P_O_^ML^, and the other site as the putative terminus, now designated P_T_^ML^, to yield the ordered pair of sites [P_O_, P_T_]^ML^. This provides a formal approach that is preferable to estimates based on graph-based approaches [16-19] particularly in cases where there is a small amount of skew that can be difficult to study by eye. Additionally, the model allows us to generate confidence intervals by a Monte Carlo method as outlined above for the ML surface.

### Software

All analyses were performed using C source code programs written and compiled for Mac OSX by RAM. A description of the three core programs used to determine model M0 parameters, the ML [Ori, Ter] location and plot a visualization of the ML surface is provided as additional material (see Additional file 3). Code is available upon request from morton@mcmaster.ca.

## List of abbreviations

ML – maximum likelihood, IG – intergenic sites, CDS – nucleotide sites in a protein-coding DNA sequence, divided into C_i _for the i^th ^codon position and further into D_4 _for four-fold degenerate C_3 _sites, R-(in)dependent – Replication arm-, DF – degrees of freedom.

## Competing interests

The author(s) declares that there are no competing interests.

## Authors' contributions

RAM wrote the computer programs to implement the model. All other contributions were equal. All authors read and approved the final manuscript.

## Supplementary Material

Additional file 1 **Summary information and data for 352 bacterial chromosomes**. Data, including the NCBI accession numbers, for each of the bacterial chromosomes used in this study. Click here for file

Additional file 2 **Additional examples of likelihood surfaces together with [G-C] and [A-T] skew walks**. Figure 1. *E. coli *[NC_000913]. Figure 2. *S. meliloti *1021 plasmid pSymA [NC_003037]. Figure 3. Nostoc sp. PCC 7120 [NC_0033272]. Figure 4. *Prochlorococcus marinus *str. MIT 9313 [NC_005071]. Click here for file

Additional file 3 **Description of computer programs**. A brief summary of the code for programs used to determine 1) model M0 parameters, 2) the ML [Ori, Ter] location and 3) plot a visualization of the ML surface. Click here for file
