# Supplementary material for: Separating the effects of mutation and selection in producing DNA skew in bacterial chromosomes
Source: BMC Genomics. 2007 Oct 12;8:369. doi: 10.1186/1471-2164-8-369 (PMC2099444; doi:10.1186/1471-2164-8-369)
Supplement: Additional file 2 — Additional examples of likelihood surfaces together with [G-C] and [A-T] skew walks. Figure 1. E. coli [NC_000913]. Figure 2. S. meliloti 1021 plasmid pSymA [NC_003037]. Figure 3. Nostoc sp. PCC 7120 [NC_0033272]. Figure 4. Prochlorococcus marinus str. MIT 9313 [NC_005071]. [file 1471-2164-8-369-S2.pdf]

Escherichia coli K12 [NC_000913]

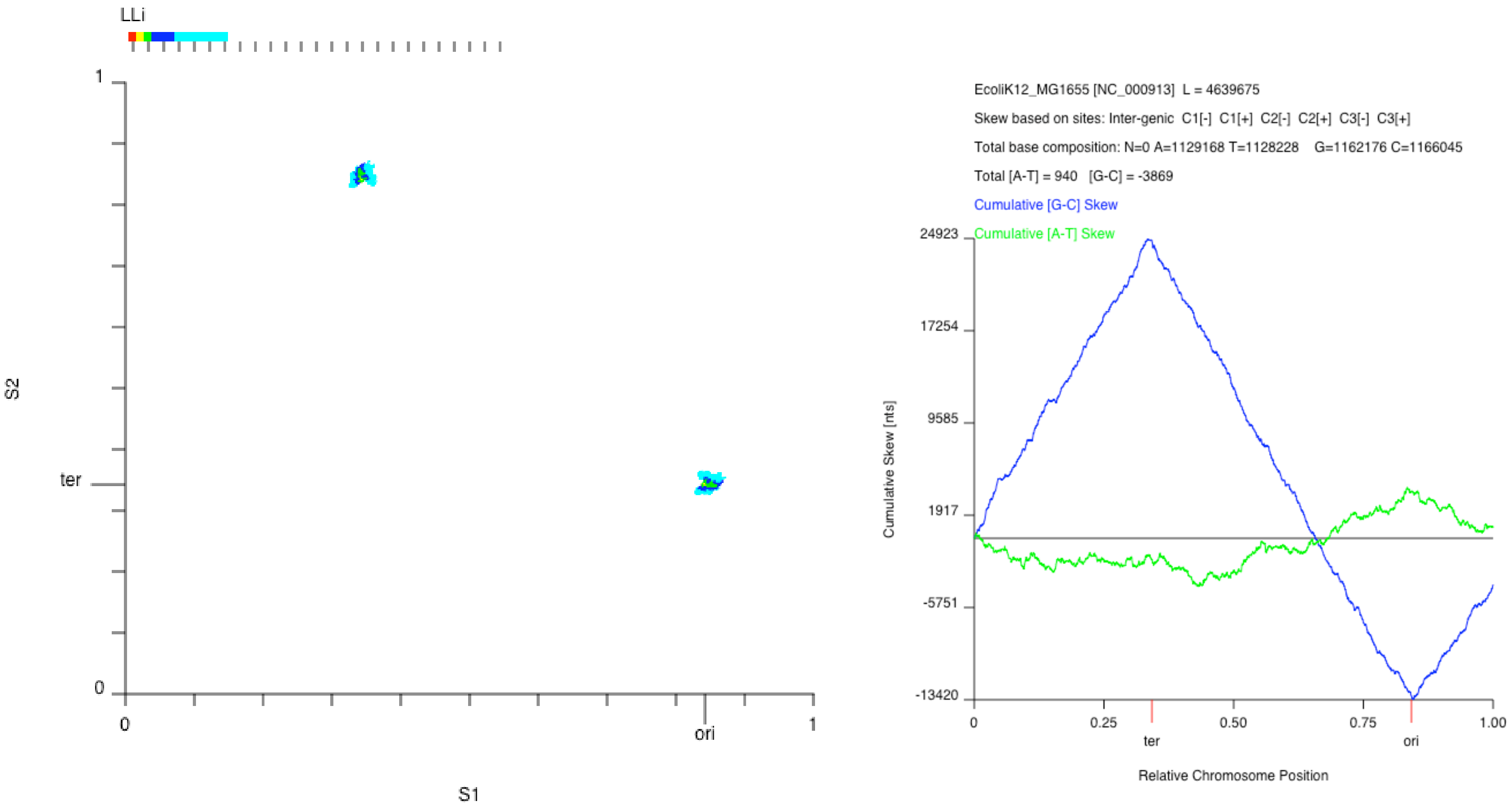

The *E. coli* chromosome has a sharp transition in GC skew at the origin and terminus. The log likelihood map has sharply defined peaks and relatively little uncertainty. R_G = 0.069 [0.064 - 0.074], R_T = 0.018 [0.012 - 0.024] (using D4 sites and the ML [O,T] location).

Sinorhizobium meliloti 1021 plasmid pSymA [NC_003037]

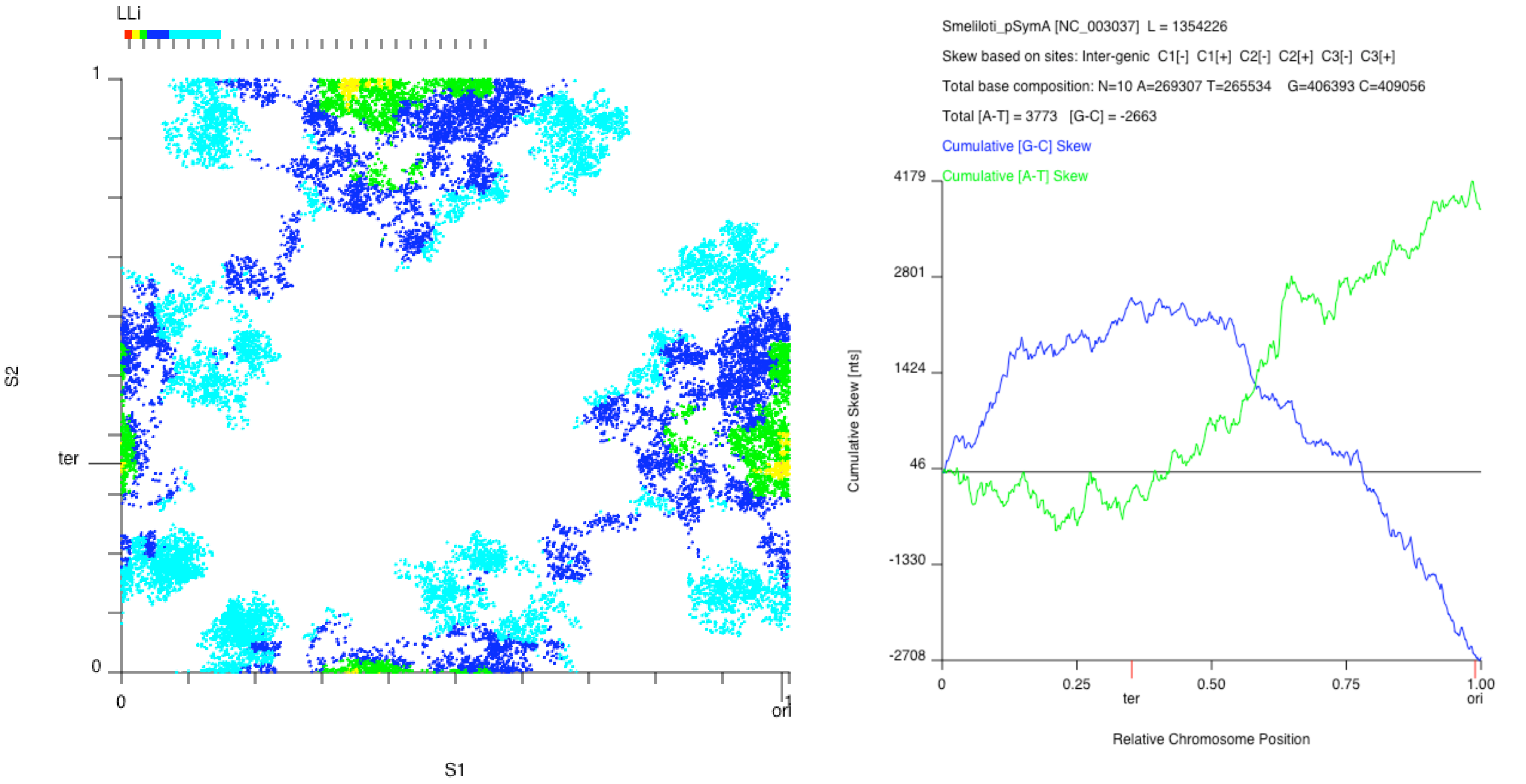

The *Sinorhizobium meliloti* 1021 plasmid pSymA chromosome has a relatively flat log likelihood surface. Nevertheless, two peaks are identified, one of them (as labeled) very close to the *repABC* genes. The chromosome walk is ambiguous, but may be used as indicative of the origin region. R_G = 0.022 [0.012 - 0.034], R_T = 0.014 [0.00 - 0.030] (using D4 sites and the ML [O,T] location).

Nostoc sp. PCC 7120 [NC_0033272]

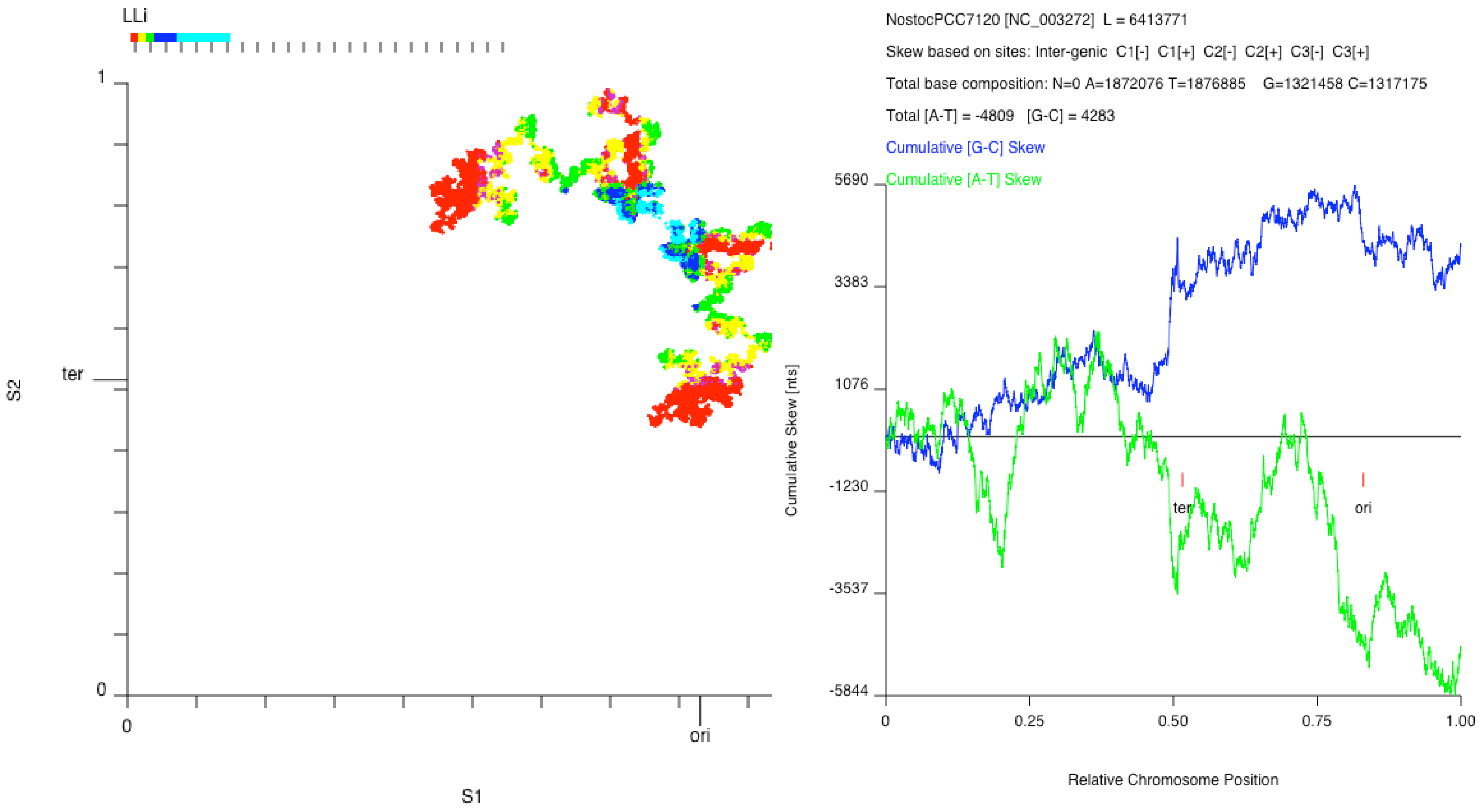

The Nostoc sp. PCC 7120 chromosome with a flat log likelihood surface. R_G = 0.001 [-0.010 - 0.012], R_T = 0.003 [-0.001 - 0.016] (using D4 sites and the ML [O,T] location).

Prochlorococcus marinus str. MIT 9313 [NC_005071]

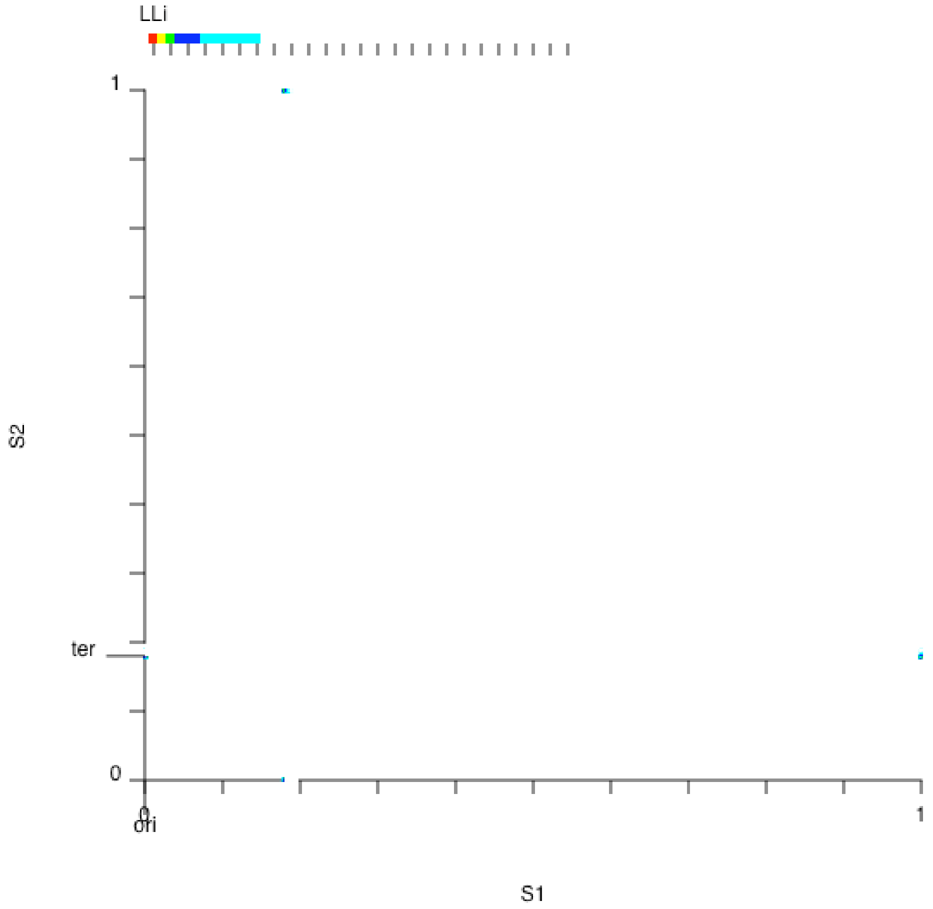

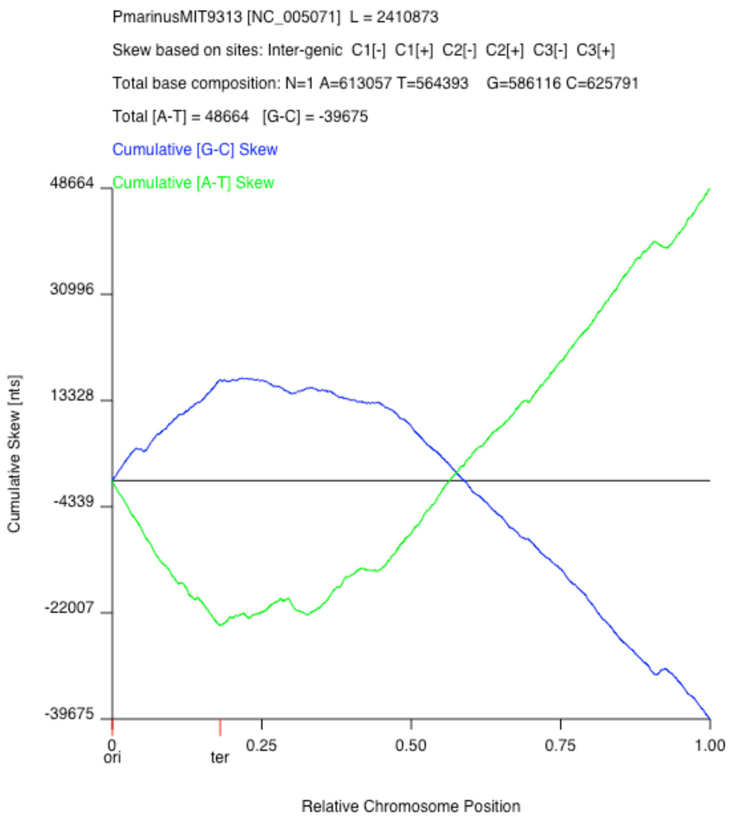

The Prochlorococcus marinus str. MIT 9313 chromosome has a sharp likelihood surface with the origin near the annotated position 1. However, the chromosome division statistic (Cd = 0.18) is small. R_G = 0.19 [0.18 - 0.21], R_T = 0.15 [0.14 - 0.16], (using D4 sites and the ML [O,T] location).
