# Supplementary material for: Separating the effects of mutation and selection in producing DNA skew in bacterial chromosomes
Source: BMC Genomics. 2007 Oct 12;8:369. doi: 10.1186/1471-2164-8-369 (PMC2099444; doi:10.1186/1471-2164-8-369)
Supplement: Additional file 3 — Description of computer programs. A brief summary of the code for programs used to determine 1) model M0 parameters, 2) the ML [Ori, Ter] location and 3) plot a visualization of the ML surface. [file 1471-2164-8-369-S3.doc]

**Description of computer programs**

Outline

**Computer**

**ML Model M0 parameter determination, SkewMLModelM0.c**

**ML [Ori , Ter] determination, SkewOTLocation.c**

**Postscript plot of [ori , ter] likelihood surface, SkewOTLikelihoodPlot.c**

**Computer**

Source code in C for Unix command line execution

Programs written using the Mac program Xcode v1.2 [Component versions Xcode IDE: 309.0 Xcode Core: 310.0 ToolSupport: 307.0]

Compiler: gcc-3.3

Run using Mac PowerPC G5 with dual 2 GHz processors and 1 GB DDR SDRAM:

Machine Model: Power Mac G5

CPU Type: PowerPC G5 (2.2)

Number Of CPUs: 2

CPU Speed: 2 GHz

L2 Cache (per CPU): 512 KB

Memory: 1 GB

Bus Speed: 1 GHz

Boot ROM Version: 5.1.8f7

Serial Number: G84362KMQPM

Operating System: OS X v10.3.9

**SkewMLModelM0**: Model M0 fit of parameters given [ori,ter] partition.

Typical run time with a single genome is ~ 2 seconds, not counting the time required to read the gene and annotation sequences into memory.

Steps Include [*E. coli K12* example]:

A) Command line defines control file: [example: modelM0_cntl_NC_000913.txt]

1) Input path to nucleotide sequence file [example: genomeseq_NC_000913.txt]

2) Input path to annotation file [example: colorseq_NC_000913.txt] which contains the annotated type of each nucleotide site interpreted according to following code:

types [strand]:

0 => "Inter-genic"

1 => "C1[-]"

2 => "C1[+]"

3 => "C2[-]"

4 => "C2[+]"

5 => "C3[-]"

6 => "C3[+]"

7 => "misc_RNA[-]"

8 => "misc_RNA[+]"

9 => "rRNA[-]"

10 => "rRNA[+]"

11 => "tRNA[-]"

12 => "tRNA[+]"

13 => "Not_Used"

14 => "ambiguous"

15 => "pseudo[-]"

16 => "pseudo[+]"

third position types [strand]:

0 => "Not_third_codon_position"

1 => "Stop[-]"

2 => "Stop[+]"

3 => "Degeneracy=1[-]"

4 => "Degeneracy=1[+]"

5 => "Degeneracy=2[-]"

6 => "Degeneracy=2[+]"

7 => "Degeneracy=3[-]"

8 => "Degeneracy=3[+]"

9 => "Degeneracy=4[-]"

10 => "Degeneracy=4[+]"

11 => "Unknown[-]"

12 => "Unknown[+]"

13 => "Degeneracy=6[-]"

14 => "Degeneracy=6[+]"

3) Chromosome length and positions of ori and ter (nts).

B) Read nucleotide sequence and annotation of nucleotide sites.

C) The genome is partition into two regions, L=ori->ter and l=ter->ori (assuming chromosome is circular). The number of nucleotides of each type is calculated for each region in the form of tables of sites and for the 3-rd position sites separately [subroutines: PartitonGenome() and Partiton3Genome()]. These partition counts are written to an output file.

D) Step over site types defined in A) using only those that are coded “on” [these are {0 to 6} for sites and {5,6,9,10,13,14} for 3-rd position sites]. Since each site contributes independently to the total likelihood, they are be maximized and parameters determined separately.

1) The five parameters for each site type for model Mobs as defined in the paper are determined from the counts in part C).

Equations:

C_A+T_ = (1/2)[(Ρ_A_ + Ρ_T_) + (ρ_A_ + ρ_T_)] (6a)

π_AT_ = (1/2)[(Ρ_A_ - Ρ_T_) + (ρ_A_ - ρ_T_)] (6b)

σ_AT_ = (1/2)[(Ρ_A_ - Ρ_T_) - (ρ_A_ - ρ_T_)] (6c)

π_GC_ = (1/2)[(Ρ_G_ - Ρ_C_) + (ρ_G_ - ρ_C_)] (6b’)

σ_GC_ = (1/2)[(Ρ_G_ - Ρ_C_) - (ρ_G_ - ρ_C_)] (6c’)

Subroutines: MobsSite() and Mobs3Site()

2) Each site used has an associated LogLikelihood (LL_k_) value determined by the equations in the paper from the partitioned nucleotide counts and the parameter values (e.g., Mobs at this stage).

Equations (for each site type k):

LL_k_ = M_A_ (LogP_A_) + M_T_ (LogP_T_) + M_C_ (LogP_C_) + N_G_ (LogP_G_)
 + N_A_ (Logρ_A_) + N_T_ (Logρ_T_) + N_C_ (Logρ_C_) + N_G_ (Logρ_G_)

where:

P_i_ = probability of nucleotide i in the region ori->ter determined from model parameters

M_i_ = number of nucleotide i in region ori->ter (data)

ρ_i_ = probability of nucleotide i in the region ter->ori determined from model parameters

N_i_ = number of nucleotide i in region ter->ori (data)

Subroutine: LogSiteLikelihood()

3) The Mobs parameter values are used as an initial guess for the simplex optimization subroutine. An initial step size is arbitrarily taken as 5%.

The simplex subroutine uses a set of 6 (5+1) parameter vectors [p(6,5)] that are initially set from the Mobs values using the step size.

The algorithm of Press et al. p.411 is used to expand or shrink the parameter simplex in an attempt to minimize –LL. Iterations are continued until the simplex size is less than a preset tolerance (1.0e-12) or a maximum number of iterations are reached (>50,000 generates error).

Typically several hundred iterations are required to reach the tolerance level.

Subroutine: MLSiteSearch()

4) The simplex optimization is repeated with the results from part 3) as the initial guess and decreasing the initial step size to 1%. The resulting parameter vector (for the site type) is the ML fit for Model M0.

5) The probability distribution for this parameter set (each site) is estimated by a [Markov Chain Monte Carlo](http://en.wikipedia.org/wiki/Markov_chain_Monte_Carlo) simulation.

a) The ML parameter vector, corresponding to the maximum likelihood, is used as the initial state.

b) Using the current (t) parameter vector with likelihood Lt, a proposal parameter vector is made by altering one parameter using a Gaussian random number with mean zero and variance sigma. [subroutine: NewGuess()]

c) The likelihood (Lp) of the proposal parameter vector is calculated for the chromosome partition. The proposal is accepted as the new state (t+1) if there is an increase in log likelihood. Otherwise, the proposal is accepted with a probability equal to Lp/Lt [10^(LLt – LLt)], using a uniform random number.

c) The value of sigma is determined by a “burn-in” procedure [subroutine BurnInSite()]. An arbitrary value of sigma is used on input and progressively decreased until the average acceptance rate is ~50% over a cycle of 100 trials.

d) Starting from the final burn-in parameter vector, a simulation of 10,000 accepted trails is made using the value of sigma from the burn-in series. [subroutine: MCMCSite()]

e) All 10,000 accepted parameter vectors are stored. On completion, these are sorted according to each parameter and the range that included 95% of the values determined for each parameter. A similar storage and sorting of the R_G_ and R_T_ values (paper, p. 17) corresponding to each accepted parameter vector is done if these statistics are desired.

6) Values for the ML parameter vectors and the 95% ranges for each site are written to the output file.

**SkewOTLocation**: Locating a ML [ori,ter] partition using Mobs parameters.

Typical run time with a single genome is ~ 2-3 hours (*E. coli* example). Longer time is required for [ori,ter] location because each new chromosome partition requires the nucleotide counts to be calculated again. The run time is very much dependent on the chromosome length. It could be substantially decreased by changing the program so that only the sites that are changed from the last [ori , ter] are recalculated.

Steps Include [*E. coli* example]:

A) Command line defines control file [example: OTlocation_cntl_NC_000913.txt]

1) Input path to nucleotide sequence file [example: genomeseq_NC_000913.txt]

2) Input path to annotation file [example: colorseq_NC_000913.txt] which contains the annotated type of each nucleotide site.

3) Chromosome length.

B) Read nucleotide sequence and annotation of nucleotide sites.

C) The basis of the procedure is the likelihood associated with an arbitrary bi-partition of the chromosome at a presumptive [ori , ter] ([o,t]) location. [subroutine:
LL = -EvaluateOT()].

1) The genome is partition into two regions, L=o->t and l=t->o. The number of nucleotides of each type is calculated for each region in the form of tables of sites [subroutine: PartitonGenome()].

2) The five parameters for each site type {IG, C1[+],[-], C2[+],[-], C3[+],[-]} for model Mobs as defined in the paper are determined from the counts in part 1) [Subroutine: MobsSite()]

3) The log likelihood of [o,t] is computed from the sum of the seven log likelihoods for each site. [Subroutine: LogSiteLikelihood()].

D) Stage 1: The likelihood for each [o,t] pair on the half grid is computed. The plane of all possible [S_1_ =>o, S_2_ =>t] values extends from o = 1 to [chromosome length] by t = 1 to [chromosome length]. It is divided into a 50x50 grid determining the grid step length in nucleotides. Since the likelihood for [o,t] = the likelihood for [t,o] (with negative σ values), likelihoods are determined for [o,t] pairs on the half plane of this grid. The bi-partition [S_1,grid_ , S_2,grid_] producing the greatest likelihood is saved for the next stage. [Subroutine: Grid()].

E) Stage 2: [S_1,grid_ , S_2,grid_] is used as an initial guess for a simplex optimization subroutine. The simplex subroutine uses a set of 3 (2+1) parameter vectors [p(3,2)] that are initially set from the [O_grid_ , T_grid_] values using the grid step length.

The algorithm of Press et al. p.411 is used to expand or shrink the [o,t] simplex in an attempt to minimize –LL. Iterations are continued until the simplex size is less than a preset toleranceor a maximum number of iterations are reached (>10,000 generates error). [subroutine SimplexOT()].

1) The first search uses a coarse initial step size and low tolerance (1.0e-6).

2) A second search begins with the [S_1_ , S_2_] obtained from the first with decreased initial step size and tolerance 1.0e-9.

3) The maximum likelihood [S_1_ , S_2_] from 2) is taken as [S_1,ML_ , S_2,ML_].

4) [S_1,ML_ , S_2,ML_] is checked for the fraction of rRNA sites on the presumed lewading strand. If this is greater than 1.0, then S_1_ is taken as ori_ML_ and S_2_ as ter_ML_, otherwise S_2_ is taken as ori_ML_ and S_1_ as ter_ML_. Ambiguous or unannotated chromosomes must be dealt with separately.

Typically a hundred iterations are required to obtain [ori_ML_ , ter_ML_].

4) The probability distribution for this parameter set (each site) is estimated by a [Markov Chain Monte Carlo](http://en.wikipedia.org/wiki/Markov_chain_Monte_Carlo) simulation.

a) The ML parameter vector [ori_ML_ , ter_ML_], corresponding to the maximum likelihood, is used as the initial state [ori_t_ , ter_t_].

b) Using the current (t) parameter vector with likelihood Lt, a proposal parameter vector is made by altering both [ori_t_ and ter_t_] using a Gaussian random number with mean zero and variance sigma.

c) The likelihood (Lp) of the proposal parameter vector is calculated for the chromosome partition. The proposal is accepted as the new state (t+1) if there is an increase in log likelihood. Otherwise, the proposal is accepted with a probability equal to Lp/Lt [10^(LLt – LLt)], using a uniform random number.

c) The value of sigma is determined by a “burn-in” procedure [subroutine BurnInSite()]. An arbitrary value of sigma is used on input and progressively decreased until the average acceptance rate is ~50% over a cycle of 100 trials.

d) Starting from the final burn-in parameter vector, a simulation of 10,000 accepted trails is made using the value of sigma from the burn-in series. [subroutine: MCMCOT()]

e) All 10,000 accepted parameter vectors are stored. On completion, these are sorted according to ori or ter and the range that included 95% of the values determined for each parameter.

f) Postscript images using the 10,000 accepted [ori , ter] pairs is made (MCMCv1.ps and MCMCv2.ps) showing the range of [ori , ter] giving “similar” likelihood values (i.e., within a preset significance range).

5) The MCMC simulation produces an [ori , ter] pair with the maximum likelihood. This is usually slightly different from that determined by the initial simplex because of jaggedness in the likelihood surface. This value is used as an initial guess in a final simplex optimization with smaller step size. The final simplex [ori_ML_ , ter_ML_] together with the 95% range from 4e) is saved as the model estimated [O,T].

**SkewOTLikelihoodPlot**: Postscript plot of the estimated [ori, ter] likelihood surface

Steps Include [*E. coli* example]:

A) Command line defines control file [example: OTplot_cntl_NC_000913.txt]

1) Input path to nucleotide sequence file [example: genomeseq_NC_000913.txt]

2) Input path to annotation file [example: colorseq_NC_000913.txt] which contains the annotated type of each nucleotide site.

3) Chromosome length and positions of ori and ter (nts). The latter are used to initiate the MCMC simulation.

B) Read nucleotide sequence and annotation of nucleotide sites.

C) Initiate a MCMC simulation as in **SkewOTLocation**. Accepted values of [S_1_ , S_2_] pairs are plotted in the [o,t] plane at two positions. The log likelihood value is converted to a color scale and plotted as a square centering on [S_1_ , S_2_] and [S_2_ , S_1_].

D) Output is saved as a postscript file (plot.ps).
